# Quantification of three-dimensional computed tomography angiography for evaluating coronary luminal stenosis using digital subtraction angiography as the standard of reference

**DOI:** 10.1186/s12938-015-0048-y

**Published:** 2015-05-30

**Authors:** Wei Guo, Xin Liu, Zhifan Gao, Sandeep Pirbhulal, Wenhua Huang, Wan-Hua Lin, Heye Zhang, Ning Tan, Yuan-Ting Zhang

**Affiliations:** Department of Cardiology, Guangdong Cardiovascular Institute, Guangdong General Hospital, Guangdong Academy of Medical Sciences, Guangzhou, China; Institute of Biomedical and Health Engineering, Shenzhen Institutes of Advanced Technology, Shenzhen, 518055 China; Key Lab for Health Informatics, Chinese Academy of Sciences, Shenzhen, 518055 China; Institute of Clinical Anatomy, Southern Medical University, Guangzhou, China; Department of Electronic Engineering, The Joint Research Centre for Biomedical Engineering, Chinese University of Hong Kong, Hong Kong, China; Shenzhen College of Advanced Technology, University of Chinese Academy of Sciences, Beijing, China

**Keywords:** Three-dimensional (3D) CT angiography, Digital subtraction angiography, Coronary luminal stenosis

## Abstract

**Objective:**

We sought to evaluate the accuracy of quantitative three-dimensional (3D) CT angiography (CTA) for the assessment of coronary luminal stenosis using digital subtraction angiography (DSA) as the standard of reference.

**Method:**

Twenty-three patients with 54 lesions were referred for CTA followed by DSA. The CTA scans were performed with 256-slice spiral CT. 3D CTA were reconstructed from two-dimensional CTA imaging sequences in order to extract the following quantitative indices: minimal lumen diameter, percent diameter stenosis (%DS), minimal lumen area, and percent area stenosis (%AS). Correlation and limits of agreement were calculated using Pearson correlation and Bland–Altman analysis, respectively. The diagnostic performance and the diagnostic concordance of 3D CTA-derived anatomic parameters (%DS, %AS) for the detection of severe coronary arterial stenosis (as assessed by DSA) were presented as sensitivity, specificity, diagnostic accuracy, and Kappa statistics. Of which vessels with %DS >50% or with %AS >75% were identified as severe coronary arterial lesions.

**Result:**

The correlations of the anatomic parameters between 3D CTA and DSA were significant (*r* = 0.51–0.74, P < 0.001). Bland–Altman analysis confirmed that the mean differences were small (from −1.11 to 27.39%), whereas the limits of agreement were relatively wide (from ±28.07 to ±138.64%). Otherwise, the diagnostic accuracy (74.1% with 58.3% sensitivity and 86.7% specificity for DS%; 74.1% with 45.8% sensitivity and 96.7% specificity for %AS) and the diagnostic concordance (k = 0.46 for DS%; 0.45 for %AS) of 3D CTA-derived anatomic parameters for the detection of severe stenosis were moderate.

**Conclusion:**

3D advanced imaging reconstruction technique is a helpful tool to promote the use of CTA as an alternative to assess luminal stenosis in clinical practice.

## Background

Atherosclerotic plaque leads to progressively increasing luminal stenosis, which could result in fatal cardiac events. Coronary angiography is currently the gold standard technique for assessment of coronary lumen stenosis or occlusion [1–4]. However, it is an invasive procedure. A catheter should be used to insert into the coronary arteries for injecting dye, which would induce discomfort for the patient. Furthermore, it has limitations of differentiating plaque components [5]. Therefore, it is of paramount importance to evaluate the lumen stenosis using non-invasive imaging techniques.

During the past decades, CT angiography (CTA) has become a rapidly developing non-invasive imaging technique, which showed promising application in the identification, visualization and characterization of the coronary artery stenosis [6, 7]. Sun et al. [8, 9] have discussed the application of 63-slice CT in the diagnosis of coronary artery stenosis intensively. Munnur et al. [10] reviewed how to identify the coronary atherosclerosis, estimate the plaque progression, assess the chest pain in the emergency department, and evaluate the functional significance of stenosis and the prognostic significance by means of CTA. Sun et al. discussed the dose reduction of CTA and the diagnostic and prognostic values on coronary artery disease. In order to evaluate the ability of CTA to identify atherosclerosis, the performance of coronary CTA has been compared to intravenous ultrasound (IVUS), IVUS with radiofrequency backscatter (IVUS/VH), single-photon emission CT (SPECT) imaging, or fractional flow reserve (FFR) in many studies [11–15]. Furthermore, more quantitative information was derived from CTA for better diagnosis of coronary artery stenosis. For example, Naganuma et al. [12] compared the quantitative measures [minimal lumen area (MLA), plaque burden, and morphology] derived from CTA to the IVUS, and FFR. One promising technique CTA-derived FFR was developed to evaluate the stenosis inside the coronary artery [16]. From one experiment of the sex differences in the visual-functional mismatch using CTA, Park et al. [17] found that female patients might have higher FFR value for any given stenosis compared with male patients. The diagnostic results of CTA could be easily affected, which produces blooming artifacts leading to high false positive rates of coronary stenosis. Sun et al. [18] tried to overcome the problem caused by the heavy calcification in the coronary artery through the measurement of left coronary bifurcation angle. More quantitative measures could be derived from the CTA data, and the clinical value of these measures was examined in 300 patients [19], or was also compared to IVUS [20]. Until now, the prognostic significance of non-invasive coronary CTA for the quantification of luminal stenosis remains controversial. Some previous works reported that anatomic measurements by noninvasive CTA have relatively poor accuracy for the quantification of stenosis severity [21, 22], and for the prediction of hemodynamically significant stenosis [21, 23]. For examples, Meijboom et al. [21] demonstrated that the correlation of the percent diameter stenosis (%DS) as determined by CTA and quantitative coronary angiography (QCA) was moderate (R = 0.53; p < 0.001), and the diagnostic accuracy (49%) was weak when CTA was used for the detection of hemodynamically significant stenosis based on FFR <0.75. Joshi et al. [22] showed that there was no relation between CTA and QCA measurements of minimal luminal diameter (MLD, r^2^ < 0.01, P = 0.57) or diameter stenosis (DS, r^2^ = 0.02, P = 0.31). However, other studies demonstrated the great diagnostic value of CTA in the assessment of luminal stenosis that was estimated by invasive imaging techniques of IVUS or catheter coronary angiography [4, 24, 25]. For examples, Youssef et al. [4] illustrated that the positive predictive value (PPV) and negative predictive (NPV) value of CTA for the detection of coronary artery stenosis based on gold standard catheter coronary angiography were 94 and 100%, respectively. Szilard Voros et al. [25] showed that the correlation of the quantitative measurements between CTA and IVUS were significant (r = 0.41–0.84, P < 0.001).

More recently, by the means of advanced imaging reconstruction technique, the evolvement of quantitative CTA measurements derived from three-dimensional (3D) coronary CTA show potential application in accurately diagnosing intermediate-to-severe coronary arterial lesions [4, 24–27], however, 3D CTA-derived luminal measurements have not been compared with digital subtraction angiography (DSA), which currently is the gold standard for luminal stenosis assessment. Therefore, the purpose of this study was to investigate the diagnostic value of 3D quantitative CTA in the assessment of coronary stenosis using DSA as the standard of reference in patients with interpretable coronary arterial stenosis.

## Methods

### Study population

The ethics committee of Guangdong General Hospital approved this retrospective study and written informed consent was obtained from all the patients or relatives before collecting their data. The data acquisition of DSA and CTA were performed in the Department of Cardiology and Department of Medical Imaging, respectively at Guangdong General Hospital. Two experienced cardiologists with 10-year experience would check the condition of each patient who was diagnosed with coronary artery disease (CAD), and suitable patients were directed to perform CTA examination. The patients with CAD confirmed by the CTA were then directed to perform DSA examination. Patients with previous coronary bypass grafts and those with coronary stents were excluded. The study population consisted of 23 patients who completed both the CTA and DSA examination from 30th August 2012 to 20th May 2014. The ages of the patients were from 42 to 81 years (62.0 ± 11.9).

### Measurements

#### CT coronary angiography

The subject was scanned with a 256-slice spiral CT (BriUiance iCT; Philips Healthcare, Cleveland, OH, USA). A bolus of contrast agent 80 mL (Ultravist 370; Schering, Berlin, Germany) and saline water 30 mL was injected intravenously at a flow rate of 4.5 mL/s. The scan started in 5 s after a threshold of 150 HU was reached in a region of interest positioned in the ascending aorta. We used a contrast agent bolus tracking method. During the scanning, prospective ECG gating was used if the patient’s heart rate was <75 beats/min, and retrospective ECG gating was used if the patient’s heart rate was >75 beats/min. Tube voltage was 120 kV, tube current was adjusted by body size [28], gantry rotation 270 ms, and pitch 0.18. Images were reconstructed at 5% interval. The reconstruction parameters were set as follows: the section thickness was 0.9 mm, reconstruction interval 0.45 mm, matrix size 512 × 512, field of view (FOV) 250 mm. Those two-dimensional (2D) imaging sequences were then transferred to another computer for 3D anatomy reconstruction.

#### Digital subtraction angiography

Invasive DSA was performed based on standard institutional protocols by X-ray angiographic equipment (Allura Xper FD10 System, Philips Healthcare, Netherland). The projection data acquisition was performed with five views of the left coronary, two views of the right coronary, and two orthogonal views of the target lesion. Once the lesion was identified from DSA data, we performed at least two sets of projection data of DSA in two orthogonal views.

#### Quantification of stenosis severity

An experienced cardiologist with 10-year experience analyzed the DSA data sets manually. Lumen segments with clearly visible coronary arterial stenosis were marked and measured by hand in the DSA images. MLD and MLA were measured in the view with the greatest degree of the stenosis. Proximal reference diameter (PRefD) and distal reference diameter (DRefD) were then measured and averaged to calculate %DS. Correspondingly, proximal reference area (PRefA) and distal reference area (DRefA) were measured and averaged to calculate percent area stenosis (%AS). %DS, %AS were calculated as the following formulas:

1 $$\% {\text{DS }} = 1 - {\text{MLD}}/[{\text{PRefD }} + {\text{ DRefD}}/2] \times 100\%$$

2 $$\% {\text{AS}} = 1 - {\text{MLA}}/[{\text{PRefA }} + {\text{ DRefA}}/2] \times 100\%$$

Another interventional radiologist with 7-year experience supervised the analysis of CTA data. The 3D CTA data were reconstructed over Mimics software (Materialise NV, Belgium) based on standard procedures as follows: First of all, coronary arterial mask construction: coronary arteries were identified from the CTA imaging sequences based on a threshold range which were set according to the different gray values of artery and other issue. Second, 3D modeling: a procedure of 3D calculating was performed with the coronary arterial masks. After that, MLD, PRefD, DRefD, MLA, PRefA and DRefA were measured in the corresponding anatomical position as marked in angiographic data sets. %DS and %AS were calculated according to the above-mentioned formulas.

### Statistical analysis

The statistical analysis was performed using SPSS (IBM Company, USA). Continuous variables were expressed as mean ± SD, while categorical variables were expressed as absolute numbers and percentages. The correlation of anatomic measurements (MLD, %DS, MLA, %AS) between CTA and DSA was assessed by Pearson correlation coefficient and linear correlation. A p value of 0.05 or less was considered statistically significant correlation. Mean differences and limits of agreement of anatomic measurements (MLD, %DS, MLA, %AS) by CTA and DSA were analyzed using Bland–Altman [29]. Since patients with severe coronary arterial stenosis were suggested to undergo intensive therapy. The diagnostic performance of 3D CTA-derived anatomic parameters (%DS, %AS) for the detection of severe coronary arterial stenosis (as assessed by DSA) was presented as sensitivity, specificity, diagnostic accuracy, positive predictive value, and negative predictive value with the corresponding 95% confidence intervals. The severity of coronary arterial lesions were classified with %DS and %AS standard, respectively. Positive was defined as vessels with severe coronary arterial stenosis (%DS ≥ 50% or %AS ≥ 75%). Negative was defined as vessels without severe coronary arterial stenosis (%DS < 50% or %AS < 75%). True positives (TP) were defined as vessels with severe coronary arterial lesions evaluated both by 3D CTA and DSA. True negatives (TN) were defined as vessels without severe coronary arterial lesions evaluated both by 3D CTA and DSA. False negatives (FN) were defined as vessels without severe coronary arterial lesions evaluated by 3D CTA-derived %AS or DS% but with severe coronary arterial lesions evaluated by DSA-derived %AS or DS%. False positives (FP) were defined as vessels with severe coronary arterial lesions evaluated by 3D CTA-derived %AS or DS% but without severe coronary arterial lesions evaluated by DSA-derived %AS or DS%. Sensitivity (Sen, true positive rate) was the calculated as TP/(TP + FN). Specificity (Spe, true negative rate) was calculated asTN/(FP + TN). Positive predictive value was calculate as TP/(TP + FP). Negative predictive value was calculate as TN/(FN + TN). The diagnostic concordance by 3D CTA-, and DSA-derived anatomic parameters for the detection of vessels with and without severe stenosis calculated as kappa statistics.

## Results

**Figure 1:**
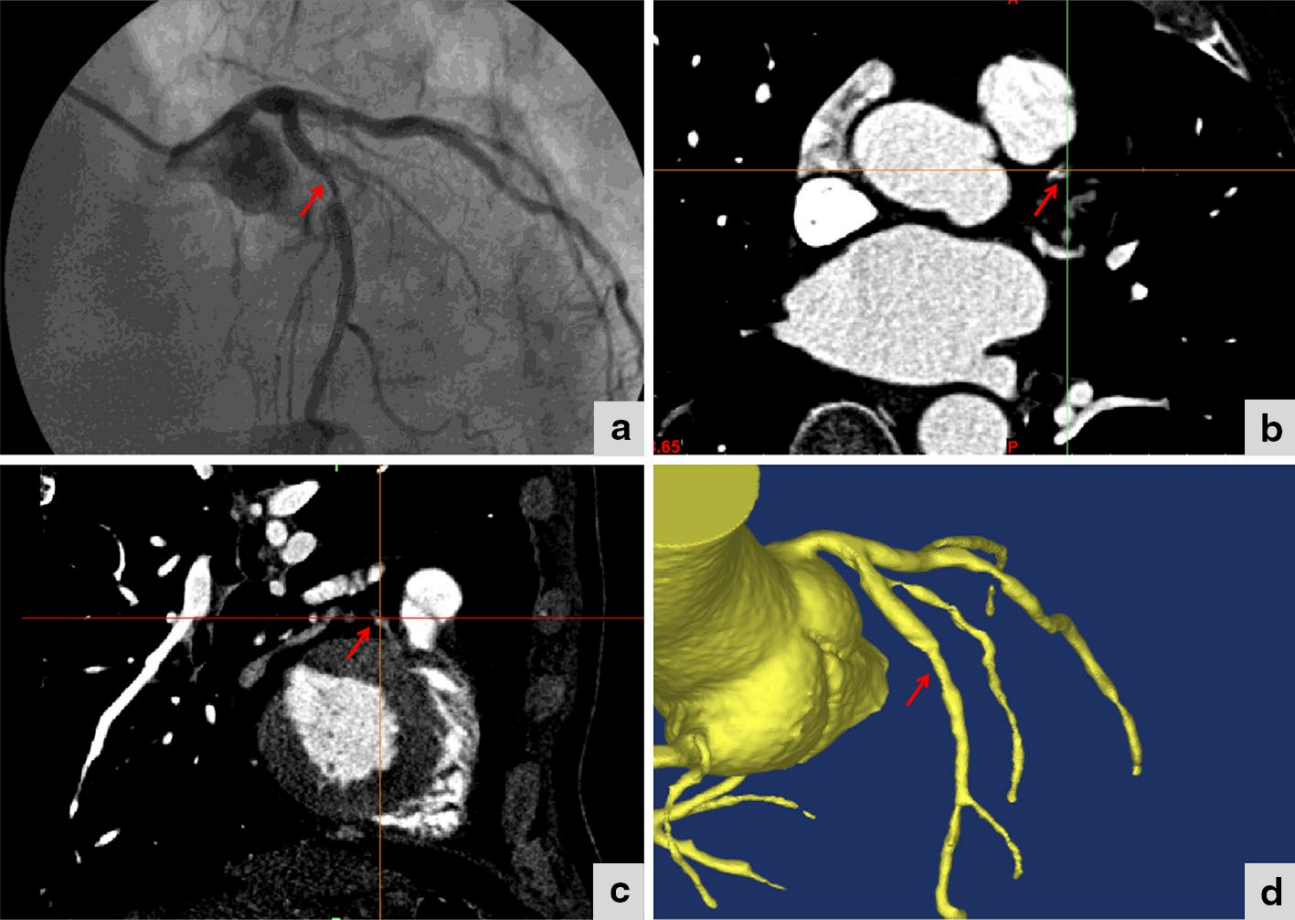
DSA, 2D CTA, and 3D CTA datasets of the same luminal stenosis. A coronary arterial segment with luminal stenosis (marked with *an arrow*) by DSA criteria is shown (**a**). Corresponding segment on 2D CTA in short axis (**b**) and in long axis (**c**) is shown at the position with the greatest degree of the stenosis. Corresponding segment on 3D CTA datasets (**d**).

General demographic features of the population are listed in Table 1. Two patients had previous myocardial infarction (MI) and no one had coronary artery bypass graft (CABG). A total 54 lesions were interpretable by both 3D CTA and DSA in the 23 patients. Distributions of the coronary lesions in the patients are also listed in Table 1. Of the 54 interpretable lesions, 26 (48.1%) lesions distributed in the left anterior descending artery (LAD), 13 (24.1%) in the right coronary artery (RCA), 8 (14.8%) in the left circumflex artery (LCX), 5 (9.3%) in the left main coronary artery (LM), 1 (1.9%) in the first diagonal (D1), and 1 (1.9%) in the first obtuse marginal (OM1). Examples of a same coronary arterial lesion detected by DSA, 2D CTA and 3D CTA are shown in Figure 1. A coronary arterial segment with luminal stenosis (marked with an arrow) by DSA criteria was shown. Corresponding segment on 2D CTA in short axis and in long axis were shown at the position with the greatest degree of the stenosis. Corresponding segment on 3D CTA datasets which were reconstructed from 2D CTA datasets by both short axis and long axis were also shown.

**Table 1:** General demographic parameters and lesions distribution

Characteristics	Frequency or value
Subjects (n)	23
Age (years)	62.0 ± 11.9
SBP (mmHg)	131.1 ± 13.6
TC (mmol/L)	4.6 ± 1.5
LDL (mmol/L)	2.7 ± 1.2
Men	13 (56.5%)
Chinese people	23 (100%)
Hypertension	6 (26.1%)
Diabetes	5 (21.7%)
Hyperlipidemia	9 (39.1%)
Smoke	6 (26.1%)
Previous MI	2 (8.7%)
CABG	0 (0.0%)
Lesions distribution	54 (100%)
LAD	26 (48.1%)
RCA	13 (24.1%)
LCX	8 (14.8%)
LM	5 (9.3%)
D1	1 (1.9%)
OM1	1 (1.9%)

*SBP* systolic blood pressure.

Lesion and reference segment characteristics and the correlation coefficients of anatomic measurements between CTA and DSA are listed in Table 2. As shown in the table, the results of Pearson correlation coefficients of anatomic measurements between CTA and DSA showed that 3D CTA-derived %DS had the strongest significant correlation with DSA (r = 0.74, P < 0.001), followed by %AS (r = 0.67, P < 0.001), and then MLD (r = 0.65, P < 0.001), and the MLA (r = 0.51, P < 0.001).

**Table 2:** Lesion and reference segment characteristics

Variables	DSA	3D CTA	Pearson correlation	P
MLD lesion (mm)	1.7 ± 0.8	1.6 ± 0.7	0.65	<0.001
Mean reference diameter (mm)	3.2 ± 0.9	2.7 ± 0.7	0.59	<0.001
%DS	47.4 ± 20.9	42.4 ± 17.4	0.74	<0.001
MLA lesion (mm^2^)	2.9 ± 2.4	3.1 ± 2.2	0.51	<0.001
Mean reference area	8.5 ± 5.1	7.2 ± 3.4	0.620	<0.001
%AS	68.1 ± 21.4	59.4 ± 19.7	0.67	<0.001

P ≤ 0.05 was considered statistically significant.

**Figure 2:**
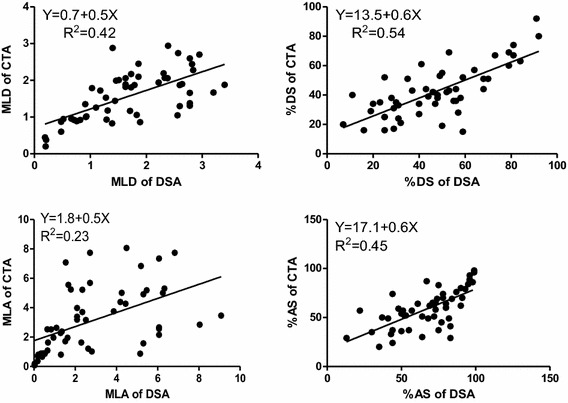
Scatterplots of the anatomic measurements (MLD, %DS, MLA, %AS) between CTA and DSA.

To illustrate the linear relationship more intuitionally, scatterplots with regression lines of MLD, %DS, MLA, and %AS between QCA and 3D CTA are shown Figure 2. The scatterplots confirmed the significant correlation between QCA and 3D CTA for the MLD, %DS, MLA, and %AS.

**Figure 3:**
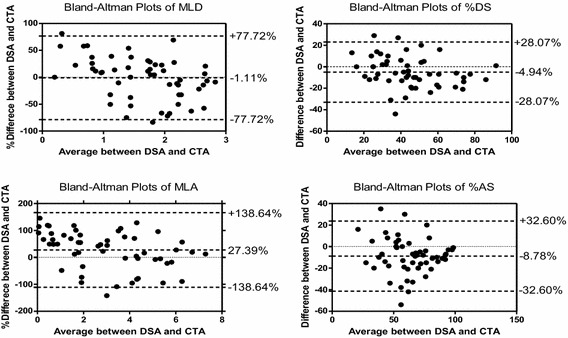
Bland–Altman plots of the anatomic measurements (MLD, %DS, MLA, %AS) between CTA and DSA.

The results of Bland–Altman analysis are shown in Table 3 and in Figure 3. The results illustrated small mean differences (−1.11, −4.94, 27.39, and −8.78% for MLD, %DS, MLA, and %AS, respectively) with relatively wide limits of agreement (±77.72, ±28.07, ±138.64, ±32.60% for MLD, %DS, MLA, and %AS, respectively).

**Table 3:** Bland–Altman analysis of the anatomic measurements between 3D CTA and DSA

Variables	Mean difference (absolute)	Limits of agreement (absolute)	Mean difference (%)	Limits of agreement (%)
MLD	−0.13	±1.29	−1.11	±77.72
%DS	−4.94%	±28.07%	−7.90	±79.05
MLA	0.26	±4.48	27.39	±138.64
%AS	−8.78%	±32.60%	−13.16	±65.03

The diagnostic performances of 3D CTA-derived anatomic parameters (%DS, %AS) for the detection of severe coronary arterial stenosis with DSA as the standard of reference are listed in Table 4.

**Table 4:** The diagnostic performances of 3D CTA-derived anatomic parameters (%DS, %AS) for the detection of severe coronary arterial stenosis (as assessed by DSA)

	TP	TN	FP	FN	Kappa	Sen (%)	Spe (%)	PPV (%)	NPV (%)	Diagnostic accuracy (%)	ROC area
%DS	14	26	4	10	0.46	58.3	86.7	77.8	91.7	74.1	0.73
%AS	11	29	1	13	0.45	45.8	96.7	72.2	69.0	74.1	0.71

The diagnostic accuracy was 74.1%, the sensitivity was 58.3%, the specificity was 86.7%, the positive predictive value was 77.8%, the negative predictive value was 91.7%, and the receiver operating characteristic curve (ROC) was 0.73 for 3D CTA-derived %DS for the detection of vessels with severe coronary arterial stenosis using DSA as the standard of reference. For 3D CTA-derived %AS, the diagnostic accuracy was 74.1%, the sensitivity was 45.8%, the specificity was 96.7%, the positive predictive value was 72.2%, the negative predictive value was 69.0%, and the ROC was 0.71. The diagnostic concordance by 3D CTA-, and DSA-derived %DS for the detection of vessels with and without severe stenosis was moderate (kappa value of 0.46); and for 3D CTA- and DSA-derived %AS, the result was also moderate (kappa value of 0.45).

## Discussion

This study was to investigate the accuracy of 3D quantitative CTA for the assessment of coronary stenosis using DSA as the standard of reference in patients with interpretable coronary arterial stenosis. The key finding of this study was that the statistical correlation between 3D CTA-derived anatomic parameters (MLD, %DS, MLA, %AS) and corresponding anatomic parameters derived from DSA were significant (r = 0.51–0.74, P < 0.001), and the Bland–Altman analysis confirmed that the mean differences was small (from −1.11 to 27.39%).

In our study, the correlations of the anatomic parameters between CTA and DSA were significant. The Pearson correlation coefficients were 0.74, 0.67, 0.65, 0.51 for %DS, %AS, MLD, and MLA, respectively. It is similar to a previous study, which also used 3D CTA technique for the quantification of coronary stenosis. They found significant correlation between CTA- and IVUS-derived anatomic parameters (r = 0.41–0.84, P < 0.001) [24]. These results are better than two previous studies, which use traditional one cross-sectional slice for stenosis analysis [21, 22]. In those studies, Meijboom et al. [21] evaluated 89 lesions in 79 patients by CTA and QCA. The subjects were scanned with a 64-slice CT scanner or a dual-source CT scanner. The correlation of the %DS as determined by CTA and QCA was significant, but moderate (R = 0.53; p < 0.001). Joshi et al. [22] evaluated 67 lesions in 55 patients by CTA and DSA. The CTA scans were performed on a 64-slice scanner. The results showed that the correlation between CTA- and QCA-derived minimal luminal diameter (MLD) or diameter stenosis (DS) were not significant. That is, for MLD, r^2^ < 0.01, P = 0.57; and for DS, r^2^ = 0.02, P = 0.31. Therefore, 3D CTA technique shows great potential for improving the accuracy of evaluating coronary stenosis. That maybe because that 3D technique can be used for the analysis of the entire vessel segment, while traditional cross-sectional slice can only provide information in two views. Another reason for the improvement of the correlation was the used of the more advanced scanner.

The diagnostic accuracy was moderate in this study (74.1% with 58.3% sensitivity and 86.7% specificity for DS%; 74.1% with 45.8% sensitivity and 96.7% specificity for %AS), which was lower than a previous study [4]. In that study, CTA image sequences were also 3D reconstructed by software, whereas the positive predictive value of CTA in detection of coronary artery significant stenosis was 94% (with 100% sensitivity and 92% specificity). That maybe because of the more advanced scanner used in that study (320-slice CT scanner). In our study, CT image series were captured by 256-slice spiral CT scanner. In addition, in our experiment, we noticed that calcifications can obscure the lumen in CTA images, which lead to the overestimation of the stenosis severity when compared with DSA. It is as similar as its influence to 2D CTA [30–32]. Therefore, further study can investigate more details of the accuracy of stenosis assessment using 3D CTA in subjects with different degrees of calcification.

A limitation of this study is that the sample size is relativity small, and all the subjects are from a single clinical center. Validation with large sample size and multi center is needed before the clinical application of using 3D CTA as an alternative to assess luminal stenosis. Another limitation of this study is that the diagnostic performance in the detection of severe coronary stenosis was moderate. That may be improved by the use of more advanced CT technique. The CT scanner is 256-slice in our study, however, more advanced CT technique is available nowadays, such as 320-slice.

## Conclusions

In conclusion, our study validated the use of noninvasive 3D CTA for anatomic assessment of stenosis. We confirmed that anatomic assessments of stenosis evaluated by 3D CTA had significant statistical correlation with those evaluated by DSA, and the mean differences were small. It shows great potential for improving the accuracy of evaluating coronary stenosis. However, the diagnostic performance of using 3D CTA for the detection of severe coronary lesion is still with respected to be improved as the improvement of spatial and temporal resolution of CT scanner. It is respected that 3D advanced imaging reconstruction technique can be a helpful tool to promote the use of CTA as an alternative to assess luminal stenosis in patients presenting with chest pain syndromes.

## Abbreviations

2D: two-dimensional
3D: three-dimensional
CTA: computed tomography angiography
DSA: digital subtraction angiography
MLD: minimal lumen diameter
MLA: minimal lumen area
%DS: percent diameter stenosis
%AS: percent area stenosis
CAD: coronary artery disease
PRefD: proximal reference diameter
DRefD: distal reference diameter
PRefA: proximal reference area
TP: true positive
TN: true negative
FP: false positive
FN: false negative
SBP: systolic blood pressure
DRefA: distal reference area
LDL: low-density lipoprotein cholesterol
TC: total cholesterol
CABG: coronary artery bypass graft
LAD: left anterior descending artery
RCA: right coronary artery
LCX: left circumflex artery
LM: left main coronary artery
D1: the first diagonal
OM1: the first obtuse marginal
ROC: receiver operating characteristic curve
Sen: sensitivity
Spe: specificity
PPV: positive predictive value
NPV: negative predictive value
MI: myocardial infarction
QCA: quantitative coronary angiography
